# A Correction Method for Wet Gas Flow Metering Using a Standard Orifice and Slotted Orifices

**DOI:** 10.3390/s21072291

**Published:** 2021-03-25

**Authors:** Barbara Tomaszewska-Wach, Mariusz Rzasa

**Affiliations:** 1 Faculty of Mechanical Engineering, Opole University of Technology, 45-758 Opole, Poland; b.tomaszewska@po.edu.pl; 2 Faculty of Electrical Engineering, Automatic Control and Informatics, Opole University of Technology, 45-758 Opole, Poland

**Keywords:** slotted orifice, wet, gas stream, stream correction

## Abstract

Flow measurements that utilize differential pressure meters are commonly applied in industry. In such conditions, gas flow is often accompanied by liquid condensation. For this reason, errors occur in the metering process that can be attributed to the fluctuations in continuous phase parameters in the flow. Furthermore, the occurrence of a dispersed phase results in flow disturbance and dynamic pressure pulsations. For the above reasons, new methods and tools are sought with the purpose of performing measurements of gas-liquid flows providing measurement results that can be considered as fairly accurate in the cases when flow involves a liquid phase form. The paper reports the results of a study involving measurement of wet gas flow using differential pressure flowmeters. The experiments were conducted for three constant mass air flow rates equal to 0.06, 0.078 and 0.086 kg/s. After stabilization of the air flow rates, water was fed into the pipe with flow rates in the range from 0.01 to 0.16 kg/s. The research involved a standard orifice and three types of slotted orifices with various slot arrangements and geometries. The analysis focused on the effect of orifice geometry on the flow metering results. On the basis of the results, it was found that the slotted orifice generates smaller differential pressure values compared to the standard orifice. The water mass fraction in the gas leads to overestimated results of measurements across the flowmeter. Regardless of the type of the orifice, is necessary to undertake a correction of the results. The paper proposes a method of gas mass flow correction. The results were compared with the common over-reading correction models available in the literature.

## 1. Introduction

Orifice meters are often applied in flow measurements involving the transport of gases in industry. In the conditions when gas is carried in industrial installations, conditions are generated that promote the condensation of liquids contained in the gas. As a result, tiny liquid droplets form, leading to errors that are attributable to the variations in the physical properties of fluids when measurements are carried out utilizing orifice meters. The reasons are associated with the loss of the homogeneous structure of the gas phase, which then takes the form of a two-phase mixture comprising gas and liquid particles. When standard methods applicable for homogeneous fluids are employed in such cases, significant error levels occur in flow metering. For the above reasons, two-phase mixture measurements form some of the major challenges in the branch of flow metrology. Such measurements normally deal with mass flow rates of gas-liquid mixtures and occur in many areas of science and technology, e.g., in petrochemical, chemical industries and in meteorology and power engineering [1,2,3]. 

Such problems are also encountered in the processes of transportation of natural gas saturated with liquid in which the volume ratio of the liquid does not exceed 10%, i.e. wet gases [3,4,5]. Due to the economic viability of extracting gases from sea bed mining sources, as well as desert, arctic and other inaccessible areas, there is a need to design and build a variety of metering systems with considerable reliability of use, coupled with small dimensions and relatively low price [4,6]. Over the past years, studies have been conducted in many research and scientific institutions with the purpose of refining the accessible techniques and developing new ones for metering two-phase mixture flows. Traditional techniques utilize equipment with large dimensions, complex design and high cost of development, hence, it is necessary to develop equipment capable of measurements with a simple design and low development costs. As a result of their simple design, low manufacturing cost and high reliability, differential pressure meters are employed as cheap and well-performing solutions for flow measurement in industrial applications. In recent studies, we can find several examples of the use of orifice meters in the applications involving measurements of mixtures consisting of gas and small ratios of the liquid phase [7,8]. 

Over the years, research has been conducted on various differential pressure flowmeter designs that could offer the possibility of reliable measurements of the flow rates of multi-phase mixtures. The directions of available studies include slotted, perforated, fractal orifices as well as orifices with different designs of holes (square, triangle, oval) [9,10,11,12,13,14]. Individual researchers supervised by Morrison, including Macek, Ihfe and Brewer, conducted experiments into the performance of slotted orifices in the conditions of single-phase as well as two-phase gas-liquid flow. These experiments demonstrated that the use of the slotted orifice leads to the homogenization of the two-phase flow when it is compared with the standard orifice. Ihfe investigated the effect of varying the porosity of the orifice along the radius of the tube and found that it is possible to obtain a fully developed flow over a short distance. The study by Macek demonstrated that the slotted orifice generates lower differential pressure and provides faster pressure recovery behind the orifice in comparison to the standard orifice [15,16,17]. Annamalai et al. [18] focused on the phenomena of homogenization of the two-phase mixture behind an orifice. In this study it was demonstrated that it is possible to apply a slotted orifice as a stream stabilizer due to its capability to transform a majority of flow structures into a homogeneous mixture. The tests examined how the homogeneity of the two-phase gas/liquid mixture varied depending on the use of the slotted orifice in a horizontal pipe for various conditions before the orifice.

## 2. Measurements of Gas-Liquid Mass Flow Rates Utilizing Orifice Plates

The flow rate measurements of fluids by application of the orifice plates involve the use of a constriction across the flow cross-section. The resulting flow interference leads to an increase in the fluid velocity. Along with the change in the velocity, the static pressure decreases and differential pressure is developed at the orifice. A reliable source applicable for determining fluid mass or volume involves the use of the differential pressure Δ*p*. It is noteworthy that orifice plates, nozzles and Venturi tubes can be listed among the variety of differential pressure flow meters operating according to this principle. [19,20]. Hence, in this work, various types of orifice meters were applied in the experimental research.

The use of orifice meters specified in the norm [19] offers the possibility of calculating mass flow rates based on differential pressure measurements. For single-phase flow, the mass flow rate can be calculated by application of the following formula:

(1) $$\dot{Q}=\frac{C}{\sqrt{1-m^{2}}}\varepsilon A_{o}\sqrt{2\Delta p\rho }$$

where *C*—discharge coefficient, *ε*—expansion factor relative to fluid compressibility, *m*—modulus of orifice, *A_o_*—area of the nozzle opening of the orifice, Δ*p*—differential pressure at orifice plate, *ρ*—fluid density.

The modulus of orifice *m* forms one of the most important quantities that characterize orifice plates. It is represented by the ratio of the surface of the orifice holes the cross-sectional area of the pipeline:

(2) $$m=\beta^{2}=\frac{A_{hole}}{A_{pipe}}$$

where *A_hole_*—area of the orifice hole, *A_pipe_*—area of pipe cross-section, *β*—beta ratio

When differential pressure flow meters are employed to measure wet gas flows, we need to bear in mind that liquid droplets in the gas flow lead to an increase in the measured differential pressure Δ*p*. This results in an overestimated gas mass flow value. To obtain accurate results, the resulting error caused by the presence of a liquid needs to be corrected. For the wet gas flow Equation (1) is corrected by the over-reading (*OR*) parameter. The *OR* parameter (3) is defined as the ratio of the apparent gas mass flow rate *Q_G,apparent_* to the mass flow rate of pure gas [21].

(3) $$OR=\frac{Q_{G,apparent}}{Q_{G}}≅\sqrt{\frac{\Delta p_{TP}}{\Delta p_{G}}}$$

(4) $$Q_{G,apparent}=\frac{C}{\sqrt{1-\beta }}\varepsilon A_{o}\sqrt{2\Delta p_{TP}\rho }$$

(5) $$Q_{G}=\frac{C}{\sqrt{1-\beta }}\varepsilon A_{o}\sqrt{2\Delta p_{G}\rho }$$

The value of *Q_G,apparent_* and *Q_G_* is derived on the basis of formulae (4) and (5), with a note that *Q_G,apparent_* represents the differential pressure for gas-liquid mixture, whereas *Q_G_* accounts for the differential pressure for the dry gas.

In order to compare the results of flow rate measurement for various orifices, modulus of orifice was adopted as the common parameter for all orifices. The use of modulus of orifice m offers a suitable representation of the similarity between the standard orifice and slotted orifices as it relates to the surface areas of the orifice. In the present research, all orifices had the same modulus. The discharge coefficient *C*—determined for the flow of incompressible fluid is characterized by the relation between the real and theoretical mass or volume flow, and for the same orifice plates its value does not depend on the installation but is only relative to the Reynolds number. The value of discharge coefficient *C* is determined experimentally for a specific type of an orifice plate.

The expansion factor *ε* [19] takes into account the compressibility of the fluid. For incompressible fluid (liquid) the expansion factor *ε* = 1, while for compressible fluids (in particular gases), its value is less than one *ε* < 1. Given the knowledge of the isentropic exponent *κ*, the expansion factor *ε* is represented by a dimensionless number that can be calculated in general form on the basis of the following formula:

(6) $$\varepsilon =\sqrt{\frac{\kappa }{\kappa -1}\frac{1}{\frac{\Delta p}{p_{L}}}{\left(1-\frac{\Delta p}{p_{L}}\right)}^{\frac{2}{\kappa }}\left[1-{\left(1-\frac{\Delta p}{p_{L}}\right)}^{\frac{\kappa -1}{\kappa }}\right]\frac{1-m^{2}}{1-m^{2}{\left(1-\frac{\Delta p}{p_{L}}\right)}^{\frac{2}{\kappa }}}}$$

For air, the isentropic exponent is equal to *κ* = 1.4.

## 3. Experimental Setup

**Figure 1:**
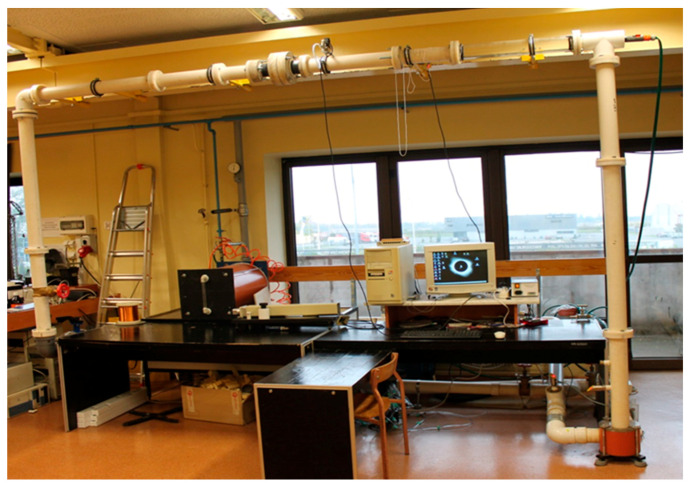
View of experimental setup.

**Figure 2:**
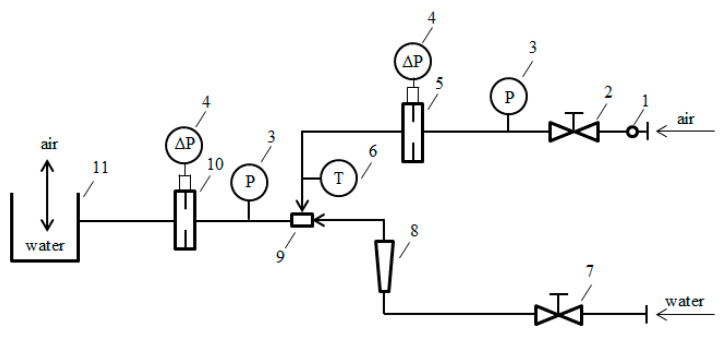
Diagram of experimental setup applied for research of flow rate of two-phase mixture: 1—pressure gauge applied to regulate air flow rate to 0.5 bar, 2—air flow rate regulation valve, 3—overpressure sensor, 4—differential pressure transducer, 5—differential pressure flowmeter applied for air flow measurements (standard orifice), 6—air temperature sensor, 7—water flow rate regulation valve, 8—water flow rate rotameter, 9—spray nozzle, 10—differential pressure flowmeter applied for two-phase flow mixture measurements (set of removable standard orifice and slotted orifices, 11—phase separator.

The experimental setup applied for testing two-phase mixture flows (see Figure 1) comprised a system of PVC pipes with a diameter of 78 mm. The experimental stand includes water and air supply systems (Figure 2). The air supply system consists of an expansion tank with a capacity of 4 m^3^ and a pressure of 0.8 MPa, a ball cut-off valve for the air supply, a pressure gauge applied for reducing the pressure to 0.5 bar. Downstream of the regulator there is a control valve that provides air flow rate regulation. The flow rate of air routed into the system is measured using a differential pressure flow meter which takes the form of a standard orifice.

In addition, the air supply system contains a pressure sensor and a temperature sensor. The water supply system consists of an installation that feeds water from the water supply network, a valve to regulate the flow rate of the liquid, a rotameter utilized for measuring the volume flow rate of water and a water-spraying nozzle which is located in a tee so that it can be installed in the experimental setup. As a result, there is a possibility of adjusting the liquid droplet size. The flow rate of the resulting two-phase mixture is measured using a differential pressure flow meter. The orifice is located in a collar with differential pressure measurements across pressure taps in an orifice plate. The differential pressure across the orifice was measured with a differential pressure transducer whose uncertainty is 5%. The remaining components of the measuring system have a negligible impact on measurement uncertainty and for this reason this aspect is disregarded in the further part of this study. For instance, the resolution of the measuring card transducer is equal to 14 bits. In the context of the measurement uncertainty, this does not have any effect on the measurements carried out using 0.10 volt signal. A similarly negligible effect is associated with the other elements of the measuring system. The two-phase mixture flows through a section in which the measurements of static pressure and temperature are additionally carried out. A glass tube has been installed on the measuring section to provide observation of the two-phase flow structure formation. A separator applied to remove liquid content the mixture from is installed at the outlet of the pipeline. The values of signals from the measuring sensors were recorded continuously on the computer throughout the experiment, and the obtained results were subsequently processed.

The experimental research was concerned with the wet gas flow rates and was carried out for three different air flow rate values of 0.06, 0.078 and 0.086 kg/s, respectively. After stabilization of the air stream, water flow rates from 0.01 to 0.16 kg/s were applied in the installation.

**Figure 3:**
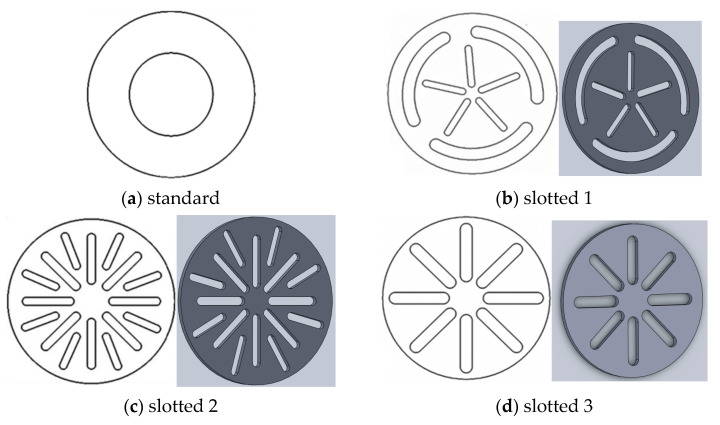
Orifice plates utilized in the experimental research.

The experiments utilized a standard orifice and three types of slotted orifices with various layouts of the perforations (Figure 3). The modulus *m* for all orifice plates was equal to *m* = 0.25. For this purpose, the size and number of perforations were selected accordingly.

## 4. Effect of Slot Geometry in Orifices on Dry Gas Flow Measurements

Experiments were carried out in the conditions characterized by wet and dry gas flows. For this purpose, the differential pressure developed across the slotted orifices applied in this study was compared. Besides, the pressure drop that occurred due to the application of slotted orifices was compared for various geometries of the perforations. Experiments were conducted for wet gas flows with the purpose of a comparison of the variations in the results recorded due to pressure pulsations in these conditions.

In metrological terms, the most important parameters include the differential pressure created at the orifice and the irreversible static pressure loss. The irreversible static pressure loss provides important insights from the technology point of view, as it represents losses accompanying the transport of gases. Therefore, most suitable conditions are ensured when this value is as low as possible. On the other hand, the high differential pressure forms a favorable case due to the sensitivity of gas flow measurements. Table 1 contains a summary of the values of differential pressure and static pressure loss for various alternatives of the applied orifice plates. The value of permanent pressure loss was determined for the dry gas mass flow rate equal to 0.045 kg/s.

The value denoted by Δ*p* represents the differential pressure developed across the orifice plate, whereas *p_L_* forms the value of permanent pressure loss that is derived on the basis of the following formula:

(7) $$p_{L}=\frac{\sqrt{1-\beta^{4}}-C\beta^{2}}{\sqrt{1-\beta^{4}}+C\beta^{2}}\Delta p$$

When identical conditions in terms of flow rates are ensured, the use of a standard orifice results in greater differential pressure across the flow meter compared to the use of the slotted orifice. For metrological reasons, this constitutes an advantage as an increase in metering sensitivity is gained in this way. However, when slotted orifices are applied, pressure recovery occurs faster than for the case of the standard orifice. Also, the permanent pressure loss is smaller in the case when slotted orifices are used. The value of the permanent pressure loss is smallest and equal to 662 Pa for the slotted orifice no. 3, whereas it is the largest for the standard orifice for which the permanent pressure loss is 855 Pa. To sum up, the best conditions characterized by differential pressure value are ensured for the case when a standard orifice is utilized. Generally, slotted orifices lead to the smaller values of differential pressure, which is mainly due to smaller gas expansibility characterized by gas flow through this type of orifice. In terms of the lowest static pressure loss, the best results are generated for the case of slotted orifice no.3, although the differential pressure for this orifice was found to be the lowest. The ratio of differential pressure to irreversible static pressure loss was calculated for comparison purposes. As indicated in Table 1, the ratio *(*∆*p/p_L_*) is very similar for all types of investigated standard and slotted orifices. Hence, the conclusion is that the gain resulting from the smaller irreversible static pressure loss is compensated by the smaller value of differential pressure.

To calculate the gas flow based on Equation (1), the coefficient *C* and factor ε need to be determined. Both of them vary depending on the type of orifice used. The values of these coefficients were determined experimentally for dry gas flow. Each of these coefficients was calculated for several gas flow rates and subsequently, the value was averaged (Table 2). The factor *ε* was calculated on the basis of the formula in Equation (6) assuming the isentropic coefficient *κ* = 1.4. In turn, the value of the discharge coefficient *C* was calculated based on Equation (1) having the input of the measured value of the gas flow rates using the flow meter marked as 5 in Figure 1 (Table 3).

The uncertainty was derived by application of the following formula:

(8) $$S=\sqrt{\frac{\sum {\left(C_{i}-\hat{C}\right)}^{2}}{n\left(n-1\right)}}$$

where *C_i_* forms the result of the measurement, $\;\hat{C}$ is the mean of all measurements, and *n* gives the total number of measurements.

## 5. Results of Experiments for Wet Gas

A number of experiments were carried out to assess the effect of water content in gas on measurement results. These experiments involved differential pressure measurements in the function of mass fraction of water for constant gas flow rates equal to 0.06 kg/s, 0.078 kg/s and 0.087 kg/s, respectively. The values of mass fractions of the liquid phase were derived by application of measurements of gas and liquid flows utilizing the relation below:

(9) $$x=\frac{Q_{L}}{Q_{L}+Q_{G}}\;\;$$

where *Q_L_* and *Q_G_* denote the mass flow rates of the liquid and gas phases, respectively.

**Figure 4:**
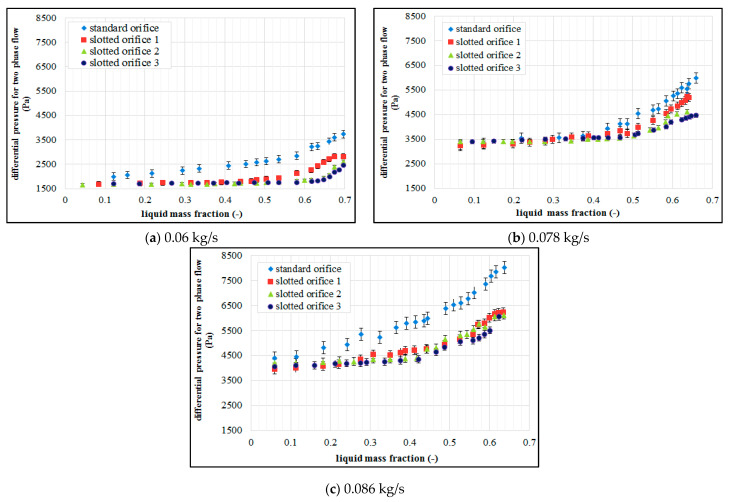
Relations between differential pressures and water mass fraction for air flow rates.

The measurements presented in Figure 4 demonstrate that the presence of liquid in the constant air flow leads to the variations in the differential pressure developed at the orifice. The analysis of the study results demonstrates that greater values of differential pressure are generated as a consequence of an increase in the mass fraction of liquid in the air flow. The distribution of the pressure relative to the mass fraction of the liquid has non-linear characteristics. When the results gained from the measurements using standard orifice are compared to the ones gained from the slotted orifice, we can see that smaller values of differential pressure are generated by the latter (in particular in the slotted orifice no. 3). A slotted orifice comprising radially arranged perforations is characterized by the smallest sensitivity of metering to the variations in the mass fractions of the liquids. This is attributable to the fact that this orifice is characterized by a steady decrease in the ratio of the diameter of perforations throughout the cross-section of the pipe. The liquid flows in an unobstructed manner through the perforations together with gas, and only a small proportion of flow is accumulated at the orifice. The arrangement of the perforations over the pipe cross-section results in the entrainment of liquid drops with the gas. In the case of the flow through the standard orifice, a considerable proportion of the liquid is held up downstream of the orifice, and liquid accumulation that leads to additional flow interference.

Figure 4 shows the results of an experiment carried out for three selected air flow rates in conditions characterized by a variable mass fraction of water. The analysis is concerned with the effect of mass fraction of water depending on the gas flow rates for selected types of orifices. On the basis of the analysis of the above figures, we can note that the presence of liquid in the gas flow results in the variations of the pressure difference developed at the orifice. We can see that the differential pressure occurring at the orifice increases along with the increase of the liquid mass flow in the conditions marked by a constant air flow rate. When an analysis of the graphs is undertaken, greater air flow rates lead to greater value in terms of the differential pressure. This tendency is recorded for each of the investigated orifice plates. The conducted experiments concerned with two-phase mixture flow across specific orifice design have demonstrated that the tested slotted orifices designs generate smaller differential pressure in relation to the standard orifice. We can see that slotted orifices no. 2 and 3 are much less sensitive in this respect. In comparison to the standard orifice, the slotted orifices demonstrate a considerably greater stability in terms of the results of measurements compared to the use of the standard orifice. On the basis of the graphs presented in Figure 4, we can see that the variations in the differential pressure depending on the mass share of the liquid in the mixture assume non-linear characteristics.

**Figure 5:**
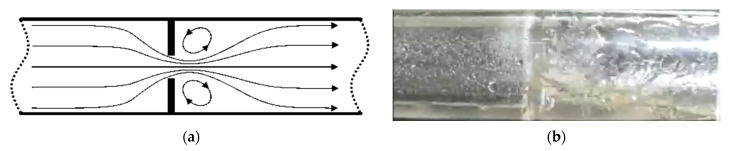
(**a**) local velocities in the fluid flow; (**b**) liquid condense on the orifice surface [22].

Apart from the sensitivity of the mass fraction of the liquid to the indications of the pressure difference for individual orifices, the phenomenon of condensation and accumulation of liquid before the orifice has a significant impact on the results of the measurements. This phenomenon was illustrated by application of the example of a standard orifice, because in this case it takes on the most intensive character (Figure 5) and involves the conditions in which local velocities in the fluid flow along the length of the contraction vary in terms of velocity and direction (Figure 5a). As a result of inertia, the liquid droplets hit the walls of the orifice and lose their momentum. The liquid condenses on the surface of the orifice and the condensed liquid falls to the bottom of the channel and is accumulated in the lowest located part of the orifice (Figure 5b). This leads to alteration in the flow geometry, which has an additional effect on the measurement results. In the case of slotted orifices, the flow is split into a series of smaller fluxes. This solution results in smaller liquid condensation on the surface of the orifice and the liquid can be much easier carried back with the gas flow. This explains why the effect of the mass fraction of liquid is much smaller in the slotted orifice than in the case of the standard orifice.

**Figure 6:**
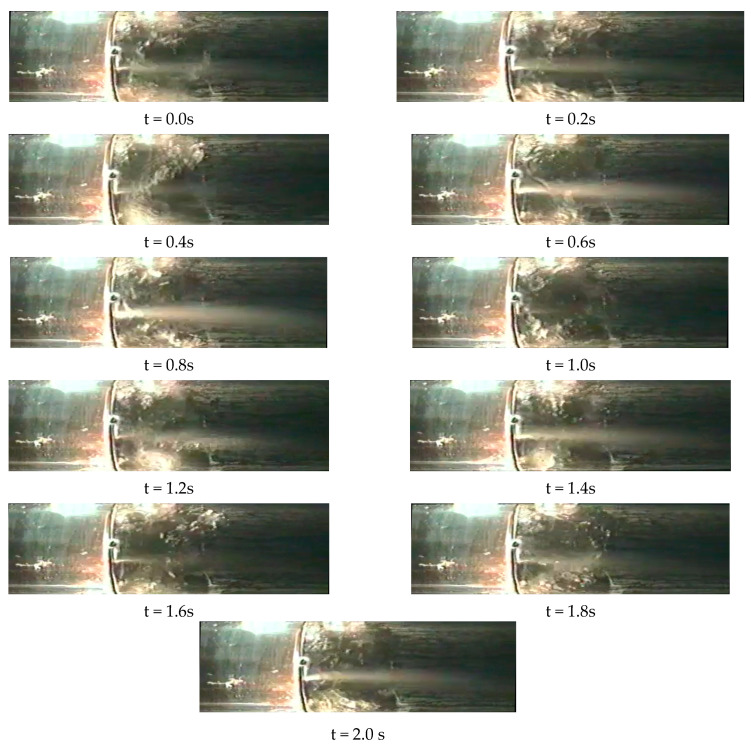
Successive images of wet gas flow through a standard orifice [23].

The accumulation of condensed liquid upstream of orifice additionally leads to pulsations of the pressure. Figure 6 contains images with the sequence of photos taken at 0.2 s time intervals. Consecutive images demonstrate fluid accumulation in front of the standard orifice. The volume of accumulated liquid increases until a critical value is exceeded. Then the liquid is suddenly lifted by the flowing gas, which forcefully moves to the other side of the orifice (t = 0.8). This type of liquid movement is accompanied by a significant increase in the measured pressure. Such pulsations have a frequency of several hertz. After such an increase in pressure, high turbulence is observed for some time, which also makes it difficult to read the measured pressure correctly. For this reason, it is recommended to use orifices for which the pulsation and stream instability will be as low as possible. Slotted orifices form a suitable solution in this respect. Therefore, they formed the subject of research in this paper. A certain difficulty is that for slotted flanges there are no standards or other documents allowing for their correct selection depending on the flow system. There is no such idea as a normalized slotted orifice for which standards would be available that would offer the basis for the calculation of parameters such as the flow coefficient, etc. Each solution requires experimental tests on the basis of which the values for the appropriate flow system are selected.

### 5.1. Correction of Gas Flow Using Over-Reading

Wet gas flow metering is associated with a number of technical obstacles [24,25]. Even inconsiderable amounts of liquid in the gas flow are known to contribute to interference in the flow measurements [1,2]. Lockhart–Martinelli parameter forms one of the most familiar parameter utilized for the purposes of determination of the relative proportion of liquid in a two-phase mixture flow. It is given by the formula in Equation (10). For wet gas flow, the *X_LM_* parameter should not exceed the value of 0.35 [4,6,26].

(10) $$X_{LM}=\frac{Q_{L}}{Q_{G}}\sqrt{\frac{\rho_{G}}{\rho_{L}}}\;\;$$

where *Q_G_* and *Q_L_* represent the mass flow rates of the gas and liquid phases, respectively, and *ρ_L_* and *ρ_G_* represent liquid and gas densities, respectively.

A variety of alternative methods are reported in the literature with the purposes of adjusting the results gained from the measurements of differential pressure flow meters relative to a parameter that characterizes wet gas [7,8]. One of the most common takes a form in which the correction is determined by a measure that expresses the response of a flow meter to the presence of the wet gas flow, coupled with variety of techniques employed to correct the results expressed by this factor. Notably, the above measure applied to determine the value of *OR* is ambiguous, as many other models are applicable in the literature available in the area of metering. A summary of the most common models can be found below.

(1) Murdock model [27]

The Murdock model is given in the form of a correlation applicable for stratified gas and liquid flow and was developed on the basis of large data sets. A suitable dependence was proposed by Murdock [27]. The use of this model is restricted only to the conditions of wet gas flow as Murdock demonstrated that such flow patterns have a considerable effect on the measurement error represented by:

(11) $$OR=1+1.26X_{LM}$$

(2) Lin model [28]

The correlation model by Lin was developed for flow through the orifice and is generally applicable for stratified flow. Lin investigated interactions at the inter-phase level. In addition, Lin proposed a variable value of the coefficient *θ_v_*.

(12) $$OR=\;1+\theta_{v}X_{LM}$$

where:

(13) $$\begin{array}{c} \theta_{v}=2.04032-1.85145\frac{\rho_{G}}{\rho_{L}}-2.24484{\left(\frac{\rho_{G}}{\rho_{L}}\right)}^{2}+9.1817{\left(\frac{\rho_{G}}{\rho_{L}}\right)}^{3}-8.42128{\left(\frac{\rho_{G}}{\rho_{L}}\right)}^{4}\\ \;\;\;\;\;\;\;\;+2.32846{\left(\frac{\rho_{G}}{\rho_{L}}\right)}^{5} \end{array}$$

(3) Chisholm model [29]

Chisholm developed an over-reading model with the purpose of characterizing two-phase flow through standard orifices. The correlation given by Chisholm accounts for some assumptions adopted for stratified flow that are expressed in terms of shear forces between phases. Consequently, it was feasible to take into account the effect of differential pressure values independently of the Lockhart–Martinelli parameter. In summary, Chisholm correlation represents the function of *X_LM_* and density of gas and liquid:

(14) OR= 1+((ρLρG)14+(ρGρL)14)XLM+XLM2

(4) Smith and Leang model [30]

The model by Smith and Leang was developed to be applicable for the standard orifice as well as for a venturi tube. It can be utilized so as to take into consideration the presence of liquid by introducing a parameter that accounts for the decrease of the pipe cross-section by the liquid:

(15) $$OR=\frac{1}{BF}$$

The parameter denoted by BF accounts for the effect of liquid in the gas flow on the cross-section area of the orifice hole. In other words, the value of parameter BF expresses the degree of the orifice cross-section blockage caused by the presence of liquid. Smith&Leange’s dependence offers a correction of the error related to measurement of gas flow rate by accounting for the gas mass fraction (1 − *x*):

(16) $$BF=0.637+0.421\left(1-x\right)-\frac{0.00183}{{\left(1-x\right)}^{2}}$$

where 1 − *x* is the gas mass fraction.

Besides, the literature contains correlations applicable for calculations of *OR* based on studies by Steven and De Leeuw, however, they are applicable for Venturi tubes; therefore, such formulae are not analyzed in this study. 

### 5.2. Method Applied for Correction of Flow Rate Measurements 

The authors of this study have developed a model using a proposition that the over-reading factor applied for the correction of the calculated air flow rate is not determined. The proposed model is applied for the correction of the differential pressure value measurement Δ*p_TP_* so that the value of the differential pressure applicable to dry gas flow Δ*p_G_* is derived with regard to the flow rate of gas occurring in the wet gas. This model is based on calculating a value of the differential pressure that would occur at the orifice if the conditions involved dry gas flow. On the basis of the experimental data, an empirical relation was developed for each of the investigated orifices. The correction method is discussed by application of the example of the standard orifice [31].

**Figure 7:**
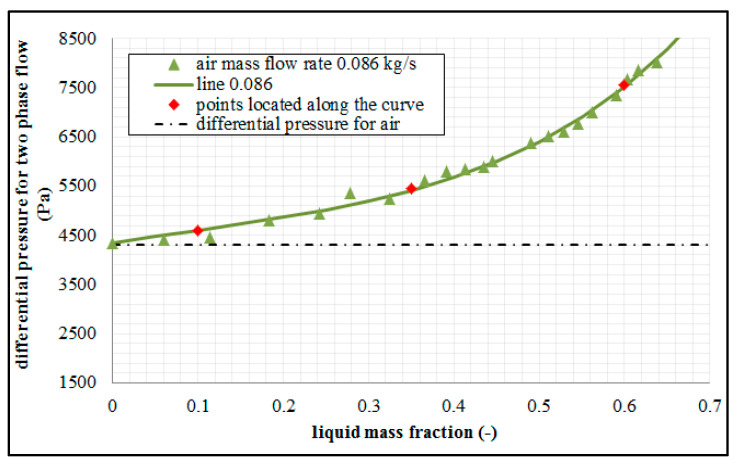
Dependence of differential pressure depending on mass fraction of liquid in the air flow.

Figure 7 contains the results of the dependence of the differential pressure on the mass fraction of liquid for the constant mass air flow rate of equal to 0.086 kg/s. The dashed line indicates the differential pressure value for dry gas. 

On the basis of Figure 7 we can see that the value of the measured differential pressure is relative to the mass fraction of the liquid to a considerable extent; and for this reason, the new model was applied with the purpose of developing a new dependence.

The measuring points were approximated by a line that can be defined by a mathematical function. As it was demonstrated throughout the experiment, the best approximation was obtained for the function expressed by a formula whose general form is given in the following form:

(17) $$\Delta p_{TP}=A_{k}\left(\Delta p_{G}\right)\cdot x^{4}+B_{k}\left(\Delta p_{G}\right)\cdot x+C_{k}\left(\Delta p_{G}\right)$$

For selected results corresponding to various flow rates, the specific values of *A_k_*, *B_k_* and *C_k_* factors are derived on the basis of the solution to a system of equations. In this system, the respective values Δ*p_TP_*_1_, Δ*p_TP_*_2_ and Δ*p_TP_*_3_ as well as *x*_1_, *x*_2_ and *x*_3_ denote the coordinates of the points located along the curve that approximates the measurement points:

$$\{\begin{array}{c} \Delta p_{1}=A_{k}\left(\Delta p_{G}\right)\cdot x_{1}^{4}+B_{k}\left(\Delta p_{G}\right)\cdot x_{1}+C_{k}\left(\Delta p_{G}\right)\\ \Delta p_{2}=A_{k}\left(\Delta p_{G}\right)\cdot x_{2}^{4}+B_{k}\left(\Delta p_{G}\right)\cdot x_{2}+C_{k}\left(\Delta p_{G}\right)\\ \Delta p_{3}=A_{k}\left(\Delta p_{G}\right)\cdot x_{3}^{4}+B_{k}\left(\Delta p_{G}\right)\cdot x_{3}+C_{k}\left(\Delta p_{G}\right) \end{array}$$

where: *x*—is the liquid mass fraction.

**Figure 8:**
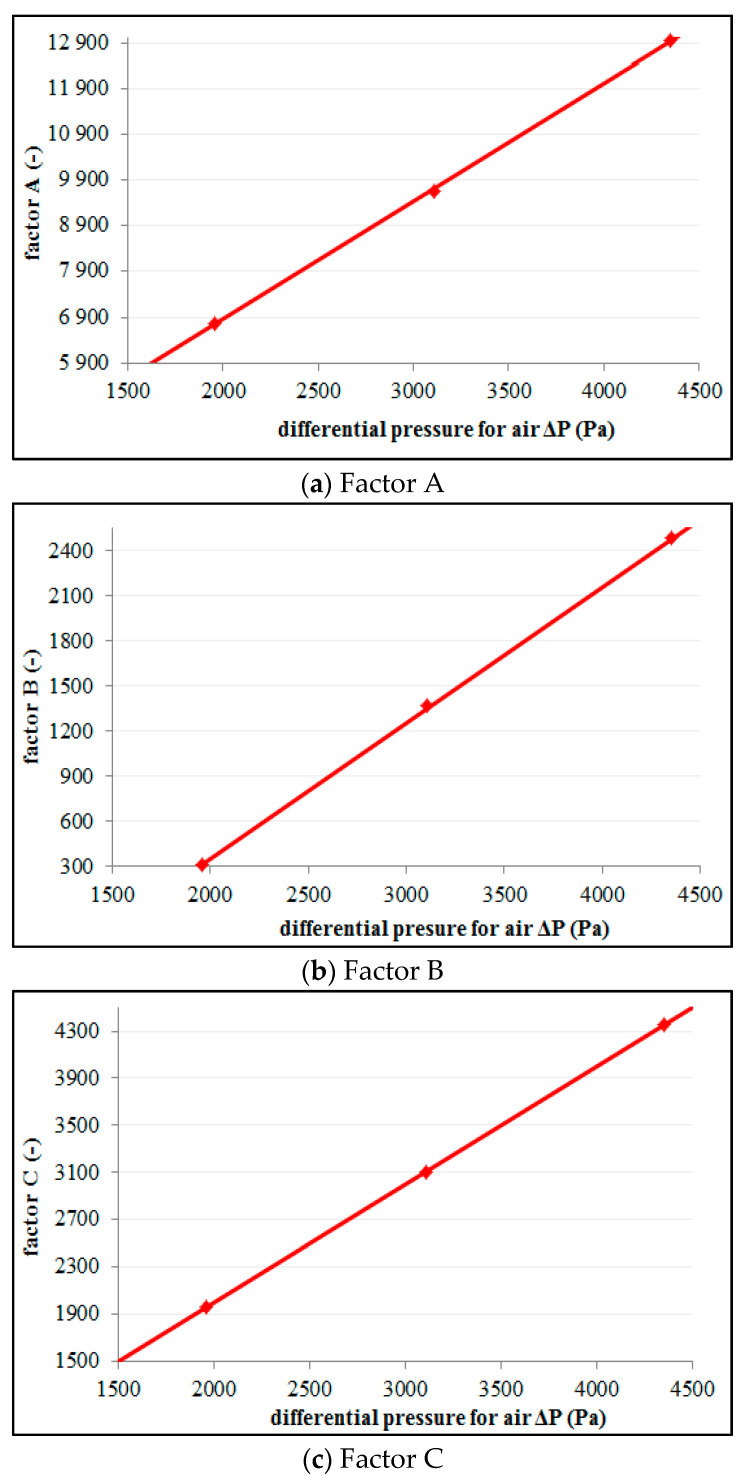
Relation between A, B and C factors and pressure for dry air.

Factors *A_k_*, *B_k_* and *C_k_* are functions dependent on the gas flow rates, and are proportional to the differential pressure values for dry gas. Therefore, several different values of *A_k_*, *B_k_* and *C_k_* need to be determined for various gas flow rates. Figure 8 contains the results of the dependence of the variations in the values of *A_k_*, *B_k_* and *C_k_* factors depending on the value of the differential pressure corresponding to various flow rates of gas. The equations were developed on the basis of the experimental data obtained for an air-water mixture [31].

On the basis of the curves in Figure 8, it was found that a good approximation is offered by a straight line that approximates the experimental points. On this basis, the values of the *A_k_*, *B_k_* and *C_k_* factors can be expressed using adequate functions. These functions express the relations between these factors and the differential pressure that is developed across the orifice for dry gas:

(18) $$\{\begin{array}{c} A_{k}=2.59\cdot \Delta p_{G}+1650\\ B_{k}=0.905\cdot \Delta p_{G}-1460\\ C_{k}=\Delta p_{G\;} \end{array}$$

For example, for a wet gas flow rate with a mass fraction of water *x*, which leads to the development of the differential pressure Δ*p_TP_* on a given orifice for a given flow rate, the value of differential pressure that would be developed on the same orifice for dry gas is equal to Δ*p_G_*. After we substitute the relation (18) in the place of Equation (17), it will take the following form:

(19) $$\Delta p_{TP}=\left(2.59\cdot \Delta p_{G}+1650\right)\cdot x^{4}+\left(0.905\cdot \Delta p_{G}-1460\right)\cdot x+\Delta p_{G}$$

In practice, it is often more suitable to apply the Equation (19). The reason is associated with the fact that as a consequence of this procedure, an equation is derived in which the only unknown is the value of the differential pressure Δ*p_G_* that is formed at the orifice for dry gas with the same flow rate. By solving this equation, the differential pressure Δ*p_G_* can be suitably calculated. On this basis, the mass air flow is calculated by inserting the value of Δ*p_G_* into Equation (1). 

A similar procedure was applied to determine the values of *A_k_*, *B_k_* and *C_k_* factors for slotted orifices. To provide the details along with the values of correction factors, a summary of the results is given in Table 4 with regard to the standard and individual design of slotted orifices.

**Figure 9:**
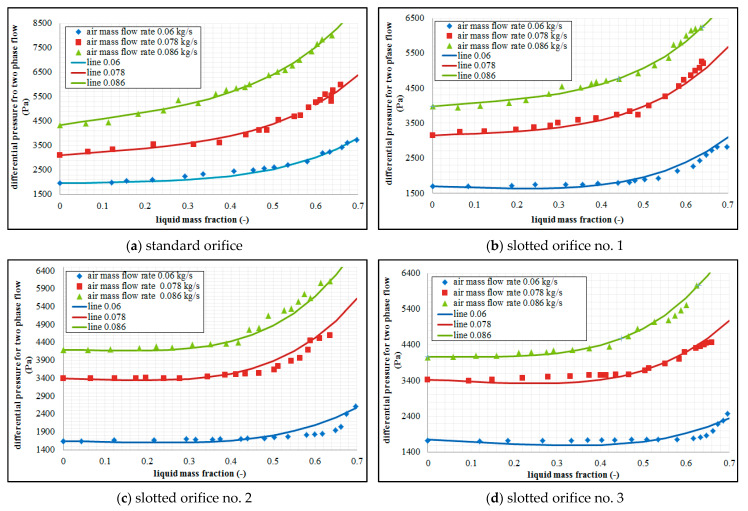
Dependence of differential pressure on the liquid mass fraction.

Figure 9 contains details of the comparison of the results of the calculated differential pressure Δ*p_TP_* derived on the basis of the relationship (19) with the data with regard to the differential pressure Δ*p_TP,experiment_* derived from experiments. This set of data refers to three different constant air flow rates and all orifice designs.

As we can conclude on the basis of the measurement data, the non-linearity of the measurement curves increases with an increase in the gas flow rate. The theoretical lines 0.06, 0.078, 0.086 that were derived on the basis of the formula in (19) suitably represent the characteristics of the effect of water in the measured air flow rate on the value of the measured differential pressure. This demonstrates that the process of development of the function (19) proceeded correctly.

Following the determination of correction factors for individual types of orifice, it was possible to state a general formula for the parameter *OR*:

(20) $$OR=\sqrt{\frac{A_{k}x^{4}+B_{k}x+C_{k}}{\Delta p_{G}}}$$

## 6. Results of Flow Rate Measurement Correction

**Figure 10:**
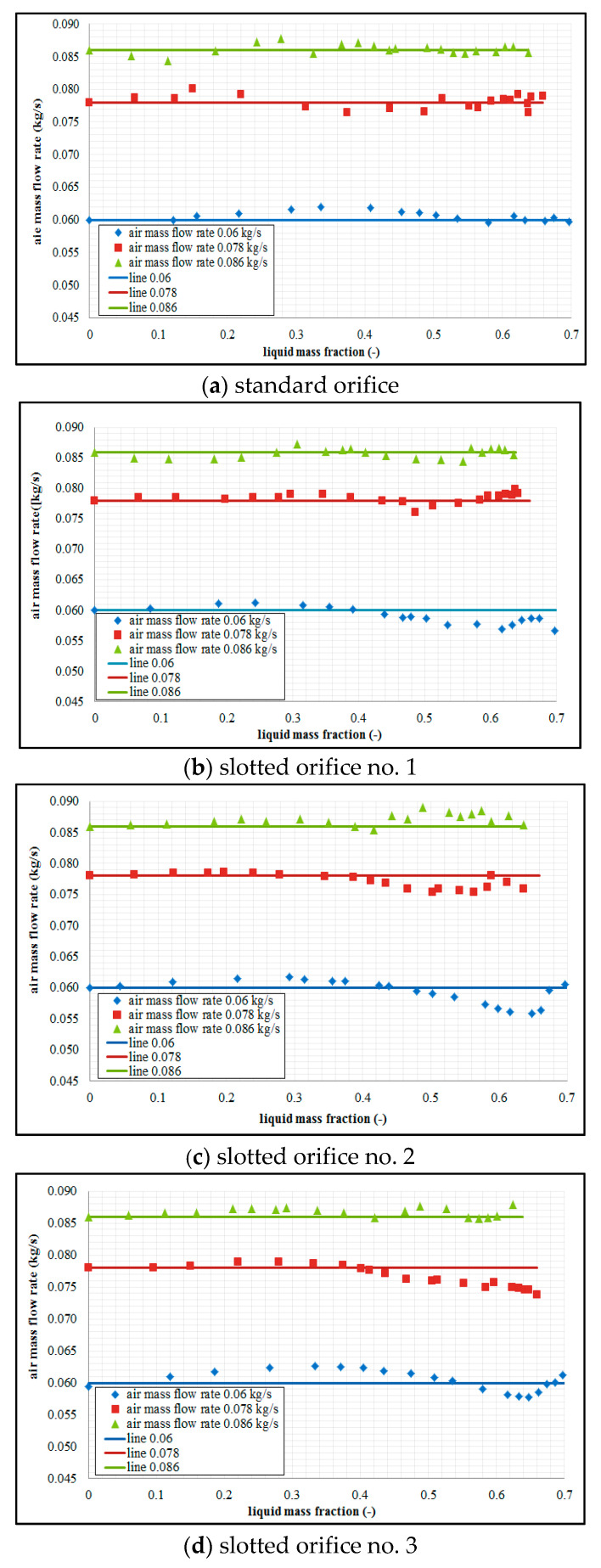
Correction of air flow rate on the basis of the model developed by authors.

Figure 10 contains the results of experimental studies using the correction developed for the standard orifice and slotted orifices.

By analyzing the curves in Figure 10, we can see that correction models proposed in this study show satisfactory conformity with the actual gas flow rates. However, these models require individual calibration for each case of measurement using an orifice.

**Figure 11:**
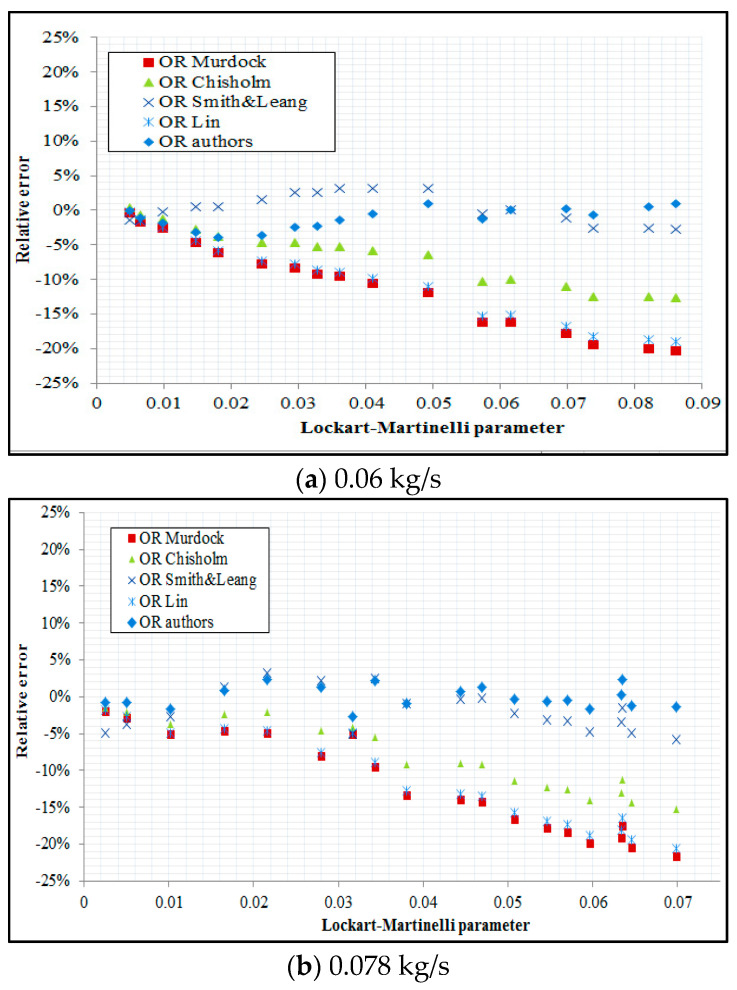

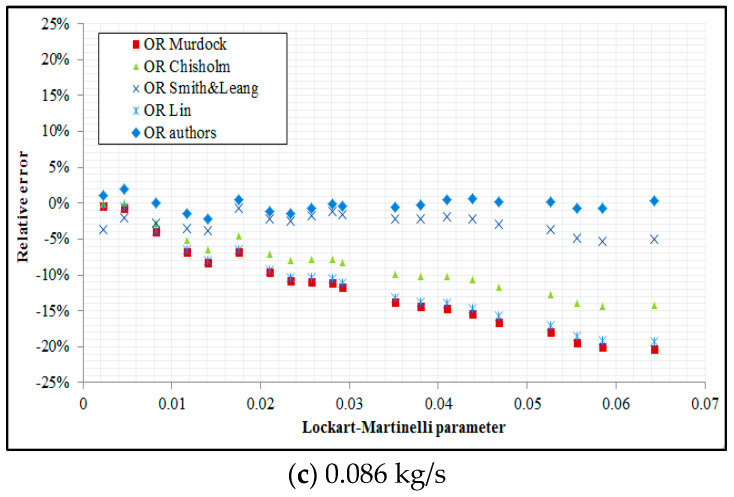
Comparison of relative error of parameter OR for various correlations applied for standard orifice in the conditions of constant air flow rates: 0.060 kg/s, 0.078 kg/s, 0.086 kg/s.

Figure 11 presents values of relative error values (*OR*) derived by application of models by Murdock, Chisholm, Smith & Leang as well as Lin and a comparison is made with the results gained on the basis of the correlation developed by the present authors. The relative error was calculated on the basis of the following formula:

(21) $$RE=\;\frac{OR_{correlation}-OR_{experiment}}{OR_{experiment}}$$

In addition, the root mean square relative error was derived from the following relation:

(22) $$R=\;\frac{1}{n}\sum_{1}^{n}\sqrt{RE_{i}^{2}}$$

In connection with the fact that the models by Murdock, Chisholm, Smith&Leang as well as Lin are applicable to standard orifices, the calculation results do not include results obtained for slotted orifices.

On the basis of an analysis of the above figures that relate to three different air flow rates, we can see that among the *OR* models available in the literature, the greatest level of conformity with the results of this research was obtained by application of Smith and Leang correlation. In the case of this correlation, the mean square error was in the range of around 3–5% for all three tested constant air flow rates. Using the Chisholm *OR* correlation, the relative error assumed a maximum of around 15%. The root mean square relative error resulting from applying the Murdock and Lin correlation is equal to approximately 19–21%. When the *OR* developed in this study was applied, the root mean square relative error was found to be 0.9–1.8%.

## 7. Conclusions

Research concerned with the flow of a two-phase gas-liquid mixture through a differential pressure flow meter demonstrates that the presence of liquid in the gas flow results in alterations of the differential pressure measured at the orifices with regard to both standard orifices and all slotted orifices. In comparison to the standard orifice designs, slotted orifices generate smaller differential pressure values. At the same time, they are more susceptible to variations in the mass fractions of liquid in the gas. The perforations in the orifice have a positive effect on the homogeneity of the flow and lead to the decrease in the formation of pulsations, and thus significantly eliminate the occurrence of an additional measurement error.

Unlike the case of the standard orifice, the perforations located on a large orifice surface make it easier for gas to carry the liquid flow smoothly, which prevents liquid condensation on the orifice surface. In the case of a standard orifice, a large proportion of liquid is permanently held-up at the orifice, which leads to the liquid accumulation before the orifice consequently leading to additional flow disturbance that interferes with the flow measurements. By substitution of a single centrally located hole by perforations with a radial arrangement near to the walls of the pipeline, the effect of accumulation of liquid in front of the orifice is eliminated. 

The analysis of experimental data demonstrates that even without the correction of measurements carried out by a slotted orifice, smaller levels of the measurement error are recorded in it compared to the standard orifice. However, for the purpose of obtaining considerable accuracy of measurements, it is necessary to use a correlation developed to correct the error resulting from the flow of the air-water mixture.

The application of the models proposed in the literature with regard to the measurements of wet gas flow gives greater ranges of the measurement error. Much better results were obtained for the model proposed by the authors of the work. Hence, a high degree of conformity of the calculations with the measurement results was obtained. However, we can note that the model developed in this work requires an experimental selection of linear coefficients of correction functions separately for each orifice design.

The paper focused on a correction method which can be combined with experimental research, in order to determine the adequate values of the flow coefficients. This method can significantly accelerate the calibration process. This is due to the fact that only three measuring points are sufficient to determine the values of the flow coefficient.

## Figures and Tables

**Table 1:** Comparison of metrological parameters of investigated orifices for dry air flow rate of Q = 0.045 kg/s.

Parameter	Standard Orifice	Slotted Orifice 1	Slotted Orifice 2	Slotted Orifice 3
Δ*p* [Pa]	1174	1087.4	962.4	910
*p_L_* [Pa]	855	792	700	662
Δ*p*/*p_L_*	1.373099	1.37298	1.374857	1.374622

*p_L_*—pressure loss behind orifice.

**Table 2:** Values of expansion factor for investigated orifices *ε*.

Gas Flow Rates	Standard Orifice	Slotted Orifice 1	Slotted Orifice 2	Slotted Orifice 3
0.045 kg/s	0.995	0.992	0.998	0.993
0.073 kg/s	0.990	0.992	0.989	0.989
0.102 kg/s	0.979	0.991	0.979	0.982

**Table 3:** Values of discharge coefficient *C* for investigated orifices

	Standard Orifice	Slotted Orifice 1	Slotted Orifice 2	Slotted Orifice 3
*C*	0.60653	0.60698	0.6070	0.6072
S(uncertainty)	9.09 × 10^−5^	1.24 × 10^−4^	4.57 × 10^−5^	1.43 × 10^−4^

**Table 4:** Values of correction factors for investigated orifices.

Orifice	*A_k_*	*B_k_*	*C_k_*
Standard	2.59 Δ*p_G_* + 1650	0.905 Δ*p_G_* − 1460	Δ*p_G_*
slotted 1	1.35 Δ*p_G_* + 4559	0.56 Δ*p_G_* + 1242	Δ*p_G_*
slotted 2	3.082 Δ*p_G_* + 319.75	0.0113 Δ*p_G_* + 296.15	Δ*p_G_*
slotted 3	3.091 Δ*p_G_* + 775.79	0.275 Δ*p_G_* + 1238	Δ*p_G_*

## Data Availability

Not applicable.

## Nomenclature

*A_hole_*	area of the orifice hole
*A_o_*	area of the nozzle opening of the orifice
*A_pipe_*	area of pipe cross-section
*BF*	blocked factor
*C*	discharge coefficient
*m*	modulus of orifice,
*OR*	over-reading
*p_L_*	pressure loss behind orifice.
*Q_G_*	gas mass flow rates
*Q_G,apparent_*	apparent gas flow rate
*Q_L_*	liquid mass flow rate
*Q*	mass flow rate
*S*	Uncertainty
*x*	liquid mass fraction
*X_LM_*	Lockhart–Martinelli parameter
*β*	beta ratio
Δ*p*	differential pressure at orifice plate,
Δ*p_G_*	differential pressure for dry gas flow
Δ*p_TP_*	differential pressure for gas-liquid flow
*ε*	expansion factor
*κ*	isentropic exponent
*ρ*	fluid density.
*ρ_G_*	gas density
*ρ_L_*	liquid density
